# Insights Into the Role of Matrix Metalloproteinases in Cancer and its Various Therapeutic Aspects: A Review

**DOI:** 10.3389/fmolb.2022.896099

**Published:** 2022-09-29

**Authors:** Sabeena Mustafa, Sheeja Koran, Lamya AlOmair

**Affiliations:** ^1^ Department of Biostatistics and Bioinformatics, King Abdullah International Medical Research Center (KAIMRC), King Saud Bin Abdulaziz University for Health Sciences (KSAU-HS), Ministry of National Guard Health Affairs (MNGHA), Riyadh, Saudi Arabia; ^2^ Laboratory of Molecular Medicine, Division of Cancer Research, Regional Cancer Centre (RCC), Medical College, Thiruvanananthapuram, India

**Keywords:** MMPs (metalloproteinases), cancer, angiogenesis, stem cells, ECM

## Abstract

Matrix metalloproteinases (MMPs) are zinc-dependent endopeptidases that regulate the turnover of extracellular matrix (ECM) components. Gross and La Piere discovered MMPs in 1962 during an experiment on tissue samples from a tadpole’s tail. Several subtypes of MMPs have been identified, depending on their substrate specificity and localization. MMPs are involved as essential molecules in multiple and diverse physiological processes, such as reproduction, embryonic development, bone remodeling, tissue repair, and regulation of inflammatory processes. Its activity is controlled at various levels such as at transcription level, pro-peptide activation level and by the activity of a family of tissue inhibitors of metalloproteinase, endogenous inhibitors of MMPs. Cancer metastasis, which is the spread of a tumor to a distant site, is a complex process that is responsible for the majority of cancer-related death It is considered to be an indicator of cancer metastasis. During metastasis, the tumor cells have to invade the blood vessel and degrade the ECM to make a path to new loci in distant places. The degradation of blood vessels and ECM is mediated through the activity of MMPs. Hence, the MMP activity is critical to determining the metastatic potential of a cancer cell. Evasion of apoptosis is one of the hallmarks of cancer that are found to be correlated with the expression of MMPs. As a result, given the importance of MMPs in cancer, we describe the role of these multifunctional enzymes MMPs in various aspects of cancer formation and their rising possibilities as a novel therapeutic target in this review. There is also a brief discussion of various types of therapeutic components and drugs that function against MMPs.

## Introduction

Matrix metalloproteinases (MMPs) are one of the most important families of proteases that act as a biological tool to cleave different components during the reconstruction of an extracellular matrix (ECM). ECM is a mechanical support to the cell and sets up to maintain the basic characteristic of the tissue. The interaction of cells with the ECM of their microenvironment determines the cell phenotype and its molecular functions. ECM is a complicated network composed of diverse biochemical components such as proteins, glycoproteins, proteoglycans, and polysaccharides (Yue, 2014). Apart from being a scaffold for cells/tissues to maintain their integrity and elasticity, ECM releases growth factors and other molecules that participate in various cellular pathways based on physiological demand (Csapo et al., 2020). Moreover, it mediates intercellular communication, signal transduction, and regulation of cellular events such as proliferation and cell death. ECM is a dynamic environment that constantly undergoes remodeling to maintain tissue homeostasis (Lu et al., 2011). ECM remodeling is an important and crucial event during normal and diseased physiological conditions. During ECM remodeling, cells undergo partial or complete degradation of different components of ECM. Degradation not only decreases the quantity of matrix proteins but also produces matrix protein degradation-derived bioactive fragments that are involved in various physiological and pathological processes (Cabral-Pacheco et al., 2020). This process of degradation is mediated by specific proteases such as MMPs that act spatially and temporally to bring about the remodeling (Kessenbrock et al., 2010).

The tight regulation of MMPs is responsible for maintaining the homeostasis of the body. Dysregulation in any regulatory mechanisms leads to aberrant expression of MMPs, which leads to different disease conditions like arthritis/osteoarthritis and fibrotic diseases as contributors to tissue destruction disease progression (Bonnans et al., 2014). Abnormal MMP expression has been observed in a variety of diseases, including neurological disorders such as Parkinson’s disease, Alzheimer’s disease, Japanese encephalitis, and glaucoma (Singh et al., 2015). MMPs also play role in diseases such as Crohn’s disease and hepatic ischemia (Ferrigno et al., 2020; Rautava et al., 2020). MMPs expression is generally very low in normal conditions, but elevated levels of MMPs are observed in different types of cancers and correlate with the enhanced proliferation and growth of tumors (Jiang et al., 2002). Inflammation is also now included in cancer hallmarks and has been found to be linked with the advancement of cancers and MMPs have been shown to influence inflammation in the tumor microenvironment in myriad ways (Coussens and Werb, 2002). Likewise, MMPs are also involved in various aspects of the development of cancer stem cells (Kessenbrock et al., 2015). In this review, we cover the involvement of the multifunctional enzymes MMPs in several facets of cancer formation, as well as the various therapeutic categories that can work against MMPs.

### Matrix Metalloproteinase in Proliferation, Invasion, and Migration

Metastasis is the dissemination of the cancer cells from one organ to another into a local or distant site, constituting more than 90% of cell death. Metastasis has a crucial role in the prognosis of the disease (Eccles and Welch, 2007). It occurs in a cascade of events, involving seven different steps: 1) detachment of cells from the primary site, 2) intravasation of cells into vascular or lymphatic channels, 3) survival of cells in the circulation, 4) adhesion into blood vessels, 5) extravasation of cells into new loci, 6) establishment of colonies in a new site, and 7) formation of tumor-specific blood vessels and angiogenesis. It is estimated that only 0.01% of cells that enter the circulation will successfully colonize in distant organs, hence considered to be a highly inefficient process (Valastyan and Weinberg, 2011). Stephen Paget proposed the “seed and soil hypothesis,” which states that the spread of tumor cells is governed by the interaction between the cancer cells (seed) and the host organ (soil). Studies show that premetastatic niches prepare the target organ to accept and form a secondary tumor (Liu and Cao, 2016; Peinado et al., 2017). Premetastatic niches are a specialized environment that favors cancer cell seeding and tumor development by containing protumor immune cells and altered ECM components.

Degradation of ECM leads to the invasion of tumor cells to promote metastasis. MMPs not only degrade the ECM components but also expose some binding sites to other receptors and release biologically active molecules (Walker et al., 2018). Invasive cancer cells form specialized F-actin-based protrusions of the plasma membrane called invadopodia to clear the path by the ECM degradation (Paz et al., 2014). Invadopodia are found in cancer cells with high metastatic potential. Studies have documented that different types of growth factors and cytokines are found to stimulate invadopodia formation. In this regard, a trans-membrane-type 1 MMP (MT1-MMP), MMP-14, accumulates in the invadopodia and facilitates the localized degradation of ECM during the intra/extravasation process (Jacob and Prekeris, 2015). In 2020 Yan et al., demonstrate that MT4-MMP regulates invadopodia formation and cell movement and enhanced cell migration and invasion Yan et al. (2020). MT1-MMP, a multifunctional enzyme, is also involved in the activation of pro-MMP-2, leading to tumor growth (Deryugina et al., 2001).

Moreover, MT1-MMP degrades MMP-8, and MMP-13 MT1-MMP degrades multiple ECM components, including collagen types I, II, and III; fibronectin; laminin-1; vitronectin; aggrecan; gelatin; α2-macroglobulin; αl proteinase inhibitor (α1Pi); and proteoglycans (Shiomi et al., 2010). Apart from degrading various ECM components, MT1-MMP may additionally release bioactive matrix fragments named matrikines, which function as extracellular modulators (Adair-Kirk and Senior, 2008).

### Role of Matrix Metalloproteinases in Epithelial–Mesenchymal Transition

Upregulated expression of MT1-MMP enhances metastasis by enhancing epithelial-to-mesenchymal transition (EMT) (Pang et al., 2016). In squamous cell carcinoma, EMT is associated with downregulation of E-cadherin (epithelial cadherin, E-cad) and upregulation of TWIST, ZEB, and zinc finger E-box-binding homeobox 1 (ZEB1) (Zhang et al., 2015). According to a study by Sato et al., MMP-2 was shown to be important in the invasive spread of ovarian cancer, while MT1-MMP was involved in both the activation and degradation of the extracellular matrix (ECM) as well as their cooperation with MMP-2 (Sato and Takino, 2010).

EMT is a highly coordinated event during which epithelial cells lose their epithelial characteristics and acquire a mesenchymal phenotype. In this process, epithelial cells undergo alterations in apical–basal polarity, disassemble their junctional structures, express mesenchyme cell proteins, acquire more spindle-shaped mesenchymal-like cells, and become migratory. This event is intrinsically linked to various processes such as embryonic development, wound healing, tissue fibrosis, and tumorogenesis. It has been considered to be an essential event in the invasion and migration of malignant cells during metastasis (Singh and Settleman, 2010). The loss of epithelial markers such as E-cad, claudins, and occludins and the rise of mesenchymal markers like vimentin, fibronectin, and N-cadherin are changes linked to EMT (neuronal cadherin, N-cad) (Lamouille et al., 2014; Nieto et al., 2016).

In cancer, EMT programs can be activated by various factors such as transforming growth factor-β (TGF-β), epidermal growth factor, and hepatocyte growth factor. Tumor hypoxia is a common feature of the microenvironment in solid tumors, which regulates the transcriptional factors such as ZEB1/2, TWIST, zinc finger protein SNAI1 (SNAIL), E2A proteins, and E2A immunoglobulin enhancer-binding factors E12/E47 (E12/E47) to downregulate the epithelial cell markers E-cad expression and induce mesenchymal gene expression.

Cadherins are transmembrane glycoproteins responsible for cell–cell adhesion and maintenance of normal tissue architecture (Oda and Takeichi, 2011). The role of different cadherins in the process of tumorogenesis has been studied extensively. “Cadherin-switch” is defined as the loss of E-cad and increased expression of N-cad during EMT, and this transition induces or enhances the metastatic potential of the tumor cells ( ). The adhesive activity of E-cadherin prevents cells in the tumor mass from dissociating from one another and therefore prevents spread into other tissues. The loss of E-cad can also result in the mislocalization of α-catenin and p120 catenin, which leads to the activation of mitogen-activated protein kinase (MAPK) pathways. E-cad thus acts as a tumor-suppressor protein (Na et al., 2020). Signaling pathways such as Wnt and TGF-β activate SNAIL and SLUG, and these molecules further regulate the “cadherin switch” by downregulating E-cad and inducing the expression of mesenchymal N-cad. N-cad stimulates cell proliferation through MAPK pathways. EMT also depends on the activity of MMPs through different mechanisms. Cells that undergo EMT can produce more MMPs and facilitate cell invasion and metastasis; the elevated levels of MMPs in turn enhance the EMT. In addition, stromal-like cells that are generated during EMT drive cancer progression *via* further MMP production (Radisky and Radisky, 2010). MMPs −1, −2, −3, −7, −9, −14, and −28 are the main MMPSs that participate in the EMT.

TWIST, a basic helix-loop-helix transcription factor have a major role in embryonic development. This gene was also found to be expressed in a number of malignancies, where it promotes the tumor initiation, its growth, and metastasis. Overexpression of TWIST induces EMT. Overexpression of twist increases the invasive and metastatic abilities of cancer cells by promoting the downregulation of E-cad and the induction of an EMT (Yang et al., 2004).

ZEB1 (also named TCF8 or DeltaEF1) is a zinc finger E-box binding homeobox 1, transcription factor that promotes tumor invasion and metastasis by inducing EMT. It induces EMT by downregulating the E-cad expression. Apart from this, ZEB1 regulates other target genes involved in tumor progression such as Lgl2, PATJ, HUGL2, and Crumbs3 (Zhang et al., 2015). ZEB1 can promote drug resistance and the survival of cancer cells (Zhang et al., 2015) According to a study, after focal ischemia, gelatinase A (MMP-2) and gelatinase B (MMP-9) activities in the human brain increase (Clark et al., 1997). It also indicates that increased levels in several forms of human malignancies are connected with a poor prognosis (Roomi et al., 2009; Kunz et al., 2016). Elevated MMP activity has been linked to a variety of pathologic diseases, and the therapeutic effect of MMP inhibitors is being investigated in a few animal models. MMP-7 mediated the conversion of E-cad into a soluble form, allowing cancer cells to dislodge from the primary tumor during the early stage of metastasis (Lee et al., 2007). In addition to the proteolytic functions, MT1-MMP controls the migration of tumor cells through non-proteolytic mechanisms (Gifford and Itoh, 2019). Overexpression of MMP-12 is positively correlated with metastasis of ovarian cancer (Zhang and Chen, 2017).

### Matrix Metalloproteinases in Angiogenesis

Cancer research is now much better at understanding the functional mechanisms that focus on cell transformation, and tumor progression and also aid in the development of new indicators and medicines (Bremnes et al., 2011). MMPs have been implicated in angiogenesis regulation as well as angiogenesis, vasculogenesis, and lymphangiogenesis in cancer (Quintero-Fabián et al., 2019). Angiogenesis is the formation of new blood vessels or capillaries from existent vasculature. Collagenases (MMP-1, 8, and 13) are the most important proteins in angiogenesis. Although this is a healing process, it begins in illnesses such as cancer. As a result, angiogenesis provides cancer cells with nutrition, resulting in tumor growth (Lugano et al., 2020). There are assays available to detect angiogenesis and biological activity. During its Phase I clinical investigation, Lockhart et al. outlined an angiogenesis assay of an MMP inhibitor (MMPI), BMS-275291 Lockhart et al. (2003). This method is widely used to assess the activity of noncytotoxic chemotherapeutic medicines as a biomarker.

Angiogenesis is regulated by a fine balance of pro and antiangiogenic molecules (Bisht et al., 2010). Disturbance in this balance and dominancy of proangiogenic factors results in “Angiogenic Switch” leading to sprouting, and proliferation of endothelial cells results in angiogenesis (Miller et al., 2009). MMP knockout mice model studies revealed that MMPs act as a critical molecule in the “Angiogenic Switch” in the growth of malignant cells. MMP-9 expression is required for the angiogenic switch, whereas MMP-2 activates endothelial cell survival and proliferation and initiates integrin signaling to support the angiogenesis thereby contributing to tumor growth (Deryugina and Quigley, 2015). In addition to its ECM degrading activity, MMPs mediate the release of potent inducers of blood vessel sprouting including vascular endothelial growth factor, basic fibroblast growth factor, and tumor necrosis factor-α (Conway et al., 2001). Moreover, MMPs are involved in the generation of angiogenic molecules such as angiostatin and endostatin from their precursors (Ghajar et al., 2008). In short, MMPs contribute to multiple events during angiogenesis.

### Matrix Metalloproteinases in Inflammation

**FIGURE 1:**
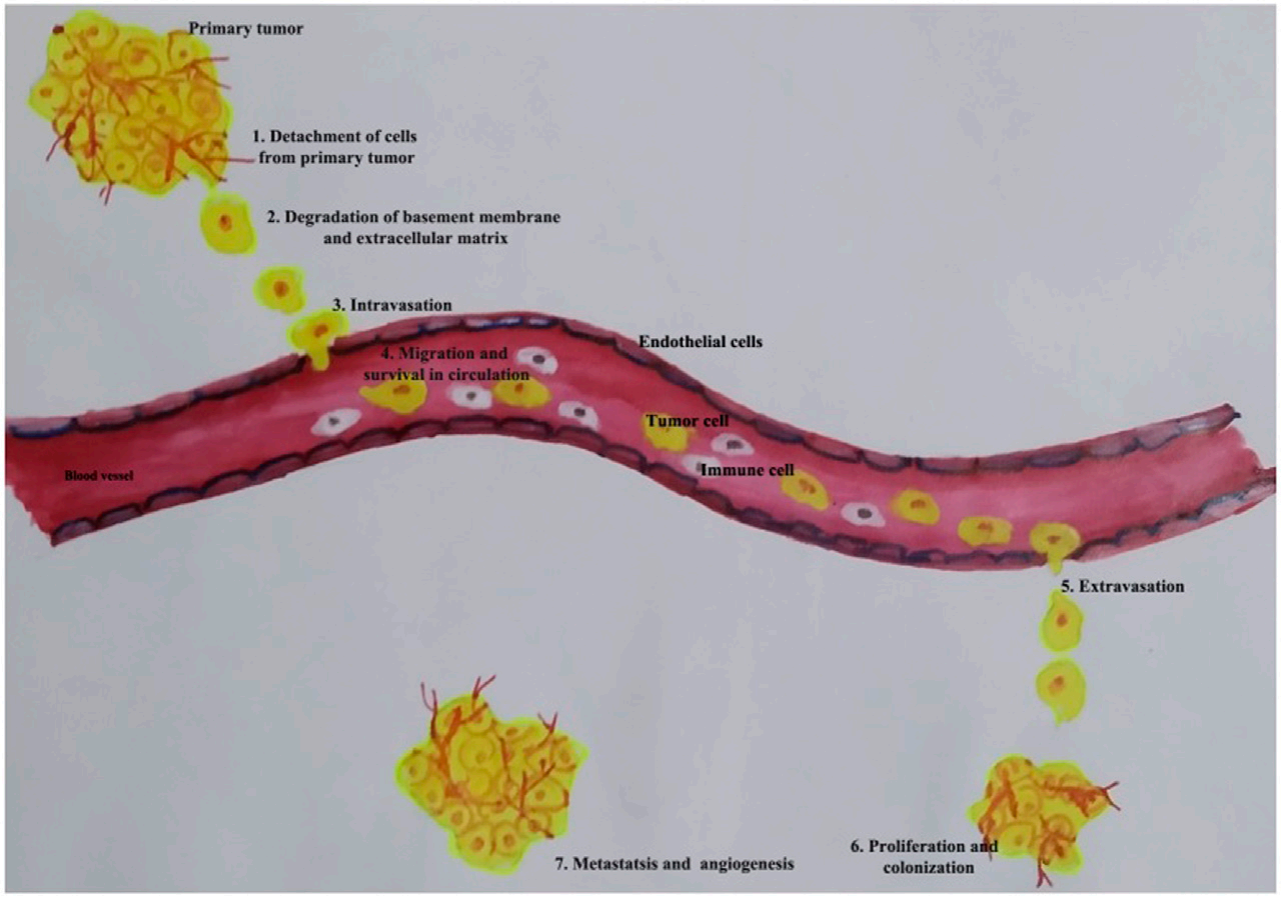
Schematic overview of the essential steps of the metastasis.

MMPs play a crucial role in cancer progression and become therapeutic targets in cancer interventions (Shay et al., 2015). MMPs are multifunctional enzymes in inflammation. Both acute and chronic inflammation can be regulated by MMP activity. Inflammation is associated with most tumor tissues and is now considered to be a hallmark of cancer linked to genetic instability (Fanjul-Fernández et al., 2010). Numerous factors, including cytokines, growth factors, chemokines, and extracellular matrix-modifying enzymes like metalloproteinases, can contribute to inflammation's ability to increase the risk of cancer (Landskron et al., 2014). Some MMPs can play both beneficial and detrimental roles at different stages. MMPs control inflammation as soluble factors, at cell surfaces and even in nuclei. In almost every human tissue, the MMP family of enzymes plays a greater role in inflammation (Fingleton, 2017). MMPs act extensively in inflammation to modulate barrier function and also play a role in cytokine and chemokine activity, which leads to the formation of chemokine gradients, as demonstrated by mouse models of human disease with targeted deletions of individual MMPs (Löffek et al., 2011). Figure 1 shows an overview of the essential steps of metastasis. Because MMPs are involved in both host defense and pathological inflammatory disease, it is critical to understand the many molecular pathways by which specific MMPs participate in normal and abnormal inflammatory processes. Understanding the pathways through which MMPs work in distinct health and disease states may lead to the development of therapeutic approaches to combat MMP-mediated diseases. The cross-link between MMPs and inflammation in tumor progression is well addressed (Quintero-Fabián et al., 2019). Overexpression of MAPK phosphatases has been demonstrated to prevent MMP promoter activation (Westermarck et al., 2001). Some members of the MMP family behave as tumor-suppressor enzymes and should therefore be regarded as anti-targets in cancer therapy (Decock et al., 2011). Many investigations have found that MMPs play an important role in tumor invasion. MMPs in cancer have been extensively investigated; nevertheless, the specific involvement of different MMPs in cancer progression may be more complex than previously thought (Mittal et al., 2016). MMP-3, MMP-7, MMP-9, and MMP-12 have been identified as inhibitors of tumor development and invasion (Table 1).

**TABLE 1:** List of matrix metalloproteinases (MMPs), their enzymatic names, major substrates, and cellular location.

MMP name	Enzymatic names	Role in stages of cancer development/Major substrates	Cellular location
MMP-1	Collagenase 1 or interstitial collagenase	Invasion/native collagens (types II > I > II, VII, VIII, X, and XI) and denatured collagens	Macrophages, lymphocytes, and vascular endothelial cells (Mach et al., 1999)
MMP-2	Gelatinase A	Angiogenesis, invasion, inflammation/native collagens (types I, II, III, IV, V, VII, X, and XI), gelatin, elastin, and fibronectin	Macrophages, lymphocytes, and endothelial cells (Oviedo-Orta et al., 2008)
MMP-3	Stromelysin-1	Inflammation/nontriple helical regions of native collagens (types III, IV, V, VII, IX, X, and XI) and gelatin	Macrophages, T-lymphocytes, and endothelial cells (Choi et al., 2019)
MMP-7	Matrilysin-1	Inflammation/nonhelical segments of native collagens (types IV, V, IX, X, and XI), gelatin, elastin, and fibronectin	Endothelial cells and macrophages (Holnthoner et al., 2006)
MMP-8	Collagenase 2 or neutrophil collagenase	Native collagens (types I > II > III, VII, and X), gelatin, fibronectin, laminin subunit gamma-2, entactin, aggrecan, tenascin, Brevican core protein precursor, myelin basic protein, and fibrinogen	Vascular smooth muscle cells, macrophages, T-cells, and vascular endothelial cells (Yang et al., 2020)
MMP-9	Gelatinase B	Inflammation, metastasis/native collagens (types I, IV, V, XI, and XIV), gelatin, elastin, vitronectin, and laminin	Macrophages, T-lymphocytes, neutrophils, and endothelial cells (Ardi et al., 2007)
MMP-10	Stromelysin-2	Inflammation/collagens (Types I, III, IV, and V), gelatin	Endothelial cells and macrophages (Orbe et al., 2009)
MMP-11	Stromelysin-3	Gelatin, fibronectin, and collagen Type IV	Smooth muscle cells, macrophages, fibroblasts and B-cells, and endothelial cells (Chen et al., 2013)
MMP-12	Macrophage metalloelastase	--------------------	Macrophage (Carmeliet et al., 1997)
MMP-13	Collagenases 3	Inflammation/native collagens (Types II > III > I, VI, VII, IX, X, and XIV), gelatin, fibronectin, laminin subunit Gamma-2	Fibroblasts and macrophages, Lymphocytes and macrophages Neutrophils (Mescher, 2017)
MMP-14	Membrane-anchored MT1-MMP	Angiogenesis/native collagens (Types I, II, and III), Gelatin, Fibronectin	Vasculardothelial cells, macrophages, and fibroblasts (Zigrino et al., 2016)
MMP-15	MT 2-MMP	Fibronectin, tenascin, entactin	Fibroblasts, leukocytes, and T lymphocytes (Edsparr et al., 2011)
MMP-16	MT3 –MMP	Collagen type III, gelatin	Leukocytes and T-lymphocytes (Bar-Or et al., 2003)
MMP-17	MT4- MMP	Gelatin, fibrin, fibrinogen, myelin basic protein	Monocytes and B-cells and fibroblast (Bar-Or et al., 2003)
MMP-19	Stromelysin-4	Native collagen type IV, gelatin	Fibroblasts T lymphocytes monocytes (Hieta et al., 2003)
MMP-21	X-MMP—(Xenopus)	Gelatin, aggrecan	Fibroblasts and macrophages (Skoog et al., 2006)
MMP-23	Cysteine Array MMP (CA-MMP) or femalysin	Gelatin, casein, fibronectin	T cells (Moogk et al., 2014)
MMP-24	MT5 –MMP	Fibronectin, gelatin, chondroitin sulphate proteoglycan	T-lymphocytes and leukocytes (Bar-Or et al., 2003)
MMP-25	MT6-MMP or leukolysin	Native collagen type IV, celatin	Monocytes and leukocytes (Pei, 1999)
MMP-26	Matrilysin-2	Native collagen type IV, gelatin, fibronectin, vitronectin, fibrinogen	Endothelial cells, fibroblasts, and macrophages (Skoog et al., 2006)

MMP-2, MMP-7, MMP-9, TIMP-1, and TIMP-2 research has received considerable attention since they play a number of roles in cancer. One study found that serum antigen concentrations of MMP-7, MMP-9, TIMP-1, and TIMP-2 were considerably higher in colorectal cancer and adenomas patients compared to controls (Barabás et al., 2021). Their data suggest that MMPs, as well as their inhibitors TIMP-1 and TIMP-2, play a crucial role in colorectal cancer. MMP-2, MMP-7, MMP-9, and TIMP-2 were also investigated in the development of the recurrent depressive disorder (Table 2). A recent study sought to establish a link between MMP-2, MMP-7, and their inhibitor, TIMP-2, in adult and pediatric cancer (Kaczorowska et al., 2020).

**TABLE 2:** Type of MMPs involved in various cancers.

S.No	Cancer type	Type of MMPs overexpressed	References
1	Breast cancer	MMP-1, 2, 8, 9, 10, 11, 12, 13, 15, 19, 23, 24, 27, and 28	Köhrmann et al. (2009)
2	Oral cancer	MMP 2, 7, and 9	Farhadi and Mohamadi, (2017)
3	Prostate cancer	MMP 2 and 9	Wilson et al. (2004)
4	Lung cancer	MMP 1, 2, 7, 9, 13, and 26	Merchant et al. (2017)
5	Liver cancer	MMP 1,3, 9, P10, 11, 13, 7, 12, and 14	Naim et al. (2017)
6	Head and neck cancer	MMP 1, 2, 3, 7, 8, 9, 10, 11, 13, and 14	Rosenthal and Matrisian, (2006)
7	Colorectal cancer	MMP 1, 2, 3, 7, 8, 9, 10, 11, 13, and 14	Said et al. (2014)

C-c motif chemokine ligand 27 (CCL27), a chemokine primarily expressed by keratinocytes, and its enhanced expression activate the extracellular signal-regulated kinase 1/2 (ERK1/2) pathway and in turn overexpress MMP-7 leads to cell invasion and migration of breast cancers (Korbecki et al., 2020). Chemokines are a class of small proteins that play an important role in leukocyte migration and invasion (Martínez-Rodríguez and Monteagudo, 2021). They have the ability to participate in tumor cell cellular proliferation and migration. CCR10 expression was discovered to be elevated in breast cancer cells, and the CCL27/CCR10 axis eventually promoted breast cancer cell invasion and migration *via* elevating MMP-7 (Lin et al., 2017). CCL27 is the ligand of CCR10 (Monteagudo et al., 2012). Likewise, in gastric cancer activated CXC motif chemokine ligand 10 (CXCL10) during inflammation enhances the invasion and migration of cells through the upregulation of MMP-2 and MMP-9 (Ren et al., 2017).

MMP-8- and MMP-9-mediated collagen breakdown generates N-acetyl-proline-glycine-proline (ac-PGP) tripeptides that bind the CXC chemokine receptor 2 (CXCR2) and trigger chemotaxis of neutrophils and increases lung metastasis (Bekaert et al., 2017). In oral squamous cell carcinoma a positive correlation was observed between MMP-7 and cyclooxygenase-2. Thus, it is well documented that MMPs regulate the inflammatory status of tumor microenvironment and facilitate the advancement of tumors (Nasry et al., 2018).

### Various Inhibitors of Matrix Metalloproteinases

MMP inhibition has been extensively investigated in cancer research. Tissue inhibitors of metalloproteinases (TIMPs) are naturally occurring proteins that inhibit MMPs specifically (Wojtowicz-Praga et al., 1997). According to studies, potent and selective MMPIs have been synthesized, and clinical trials of such synthetic MMPIs began in the 1990s and early 2000s (Brown, 1999). These studies failed because of ineffectiveness and significant. Batimastat (BB-94) and marimastat (BB-2516) are synthetic, low-molecular-weight MMPIs. They have a hydroxamate structure that mimics collagen. Batimastat was the first synthetic MMP inhibitor studied in patients with advanced cancer (Wojtowicz-Praga et al., 1997). Various investigations are now being conducted to identify potent MMP inhibitors (Hidalgo and Eckhardt, 2001; Vihinen and Kähäri, 2002; Devy and Dransfield, 2011). Selective MMP inhibition has been used with antibodies and small molecule components based on binding to protease secondary binding sites, blocking the protease active site, or preventing proMMP activation. Several of these inhibitors have just undergone clinical trials, whereas others are in advanced preclinical phases (Fields, 2019a). The humanized monoclonal antibody GS-5745, a potent and highly selective allosteric MMP9 inhibitor, has been developed for clinical trials in ulcerative colitis and colorectal cancer (Marshall et al., 2015; Fields, 2019b). However, many ongoing studies are attempting to comprehend the complexity of MMP function in many diseases (Das et al., 2021). In terms of inhibiting MMP expression through kinase pathways, it is feasible that selective pharmacologic inhibitors for specific signaling pathways (e.g., MAPK and PKC) may soon be accessible for preliminary clinical trials. This will improve outcomes in a range of illnesses, including cancer, heart disease, and neurodegenerative disease. With a greater understanding of MMP protein design, new techniques for designing MMP-targeted therapeutics have emerged.

Collagen peptidomimetics and nonpeptidomimetic MMP active site inhibitors, tetracycline derivatives, and bisphosphonates are the most commonly investigated MMP inhibitors (Hidalgo and Eckhardt, 2001). Batimastat, a hydroxamate peptidomimetic inhibitor, and marimastat were the first MMP inhibitors to be thoroughly explored. The analog of batimastat, marimastat, binds to the active site of MMPs (Chaudhary et al., 2010). Marimastat is the first orally accessible MMP inhibitor to be evaluated in humans, and it has been shown in animal models to limit the spread and progression of pancreatic cancer (Rosemurgy et al., 1999). Several nonpeptidic MMP inhibitors were also produced as part of the process of generating potential therapeutic options and determining the medicinal nature and bioavailability of peptidic medicines. Angiogenesis-promoting matrix-targeting metalloproteinases are also thought to be good therapeutic targets (Stetler-Stevenson, 1999). These mechanisms are useful in understanding how drugs work (Fields, 2019a).

To target specific MMPs, researchers are currently working to identify new molecular components from nutraceuticals, such as betulinic acid, genistein, theaflavin, myricetin, curcumin, resveratrol, matlystatin B, nicotinamide, xanthorhizzol, oleanolic acid, glycyrrhetinic acid, and catechin derivatives (Mukherjee et al., 2013). Polyphenols, monophenols, and other secondary metabolites of food and nonedible plants are among these natural compounds (Pandey and Rizvi, 2009). Synthetic MMPIs were designed to prevent tumor cell-induced changes in ECM and thereby achieve antitumor activity (Mitsiades et al., 2001). Among marine-derived MMPIs that aid to suppress MMPs, marine saccharoid MMPIs are very popular (Zhang and Kim, 2009).

### Nanodelivery System for Targeting Matrix Metalloproteinases in Cancer Treatment

Conventional cancer treatment options include radiation therapy, chemotherapy, and surgery, either alone or in combination (Baskar et al., 2012). Most of the time, these therapeutic approaches have multiple major adverse effects. Cells that rapidly proliferate may be destroyed because of a lack of specificity, resulting in immunosuppression, the development of multidrug resistance, and the growth of stem-like cells, all of which can lead to treatment failure and a low survival rate. Due to the lack of solubility, the drug will stay in circulation for a shorter amount of time, lowering the penetrance or availability of the cells and resulting in therapeutic failure. Inflammation of the digestive tract lining, alopecia (hair loss), and organ failure have all been linked to the drug. Nanotechnology’s application in cancer treatment allowed researchers to overcome many of the constraints of traditional treatments, resulting in significant advancements in cancer therapy (Jin et al., 2020). Nanotechnology makes use of nanoparticles with unique optical, magnetic, and electrical properties that are designed at the atomic or molecular level. Nanoparticles range in size from a few nanometers (nm) to several hundred nanometers (nm), depending on their intended function (Cheng et al., 2021). Nanomaterials of various sorts have been synthesized for a variety of cancer therapies. Nanoparticles may circulate more freely in the human body than larger particles (Patra et al., 2018); therefore, they could be used to deliver drugs to particular cells or tissues with a controlled release. The biophysical and biochemical properties of the targeted medications and loci being treated determine the use of an ideal nano-drug delivery technology (Rizvi and Saleh, 2018).

The targeted delivery is achieved by either passive targeting or active targeting. In active targeting, a drug is conjugated with a nanoparticle, whereas in passive targeting, it is based on enhanced permeability and retention effect (Ali et al., 2021). Thus, targeted delivery helps reduce toxicity in normal cells, protects drugs from degradation, increases half-life, etc., (Rosenblum et al., 2018).

MMPs are upregulated at all stages of expression in cancers. Different strategies have been developed to inhibit their expression and enzymatic activity. However, these inhibitors have produced serious side effects and nonspecific inhibition making other pathways or molecules involved in other pathways, which causes other pathological situations. For example, marimastat, a potent synthetic MMP inhibitor, chelates the zinc ion of the MMPs catalytic site but might also inhibit the activity of other zinc-dependent enzymes (Winer et al., 2018). In this context, nanotechnology-based approaches have been developed. The nanofiber system consisting of DOX linked to the KGFRWR peptide (an amyloid ß protein derivate) was found to reduce the tumor growth in hepatocellular carcinoma. In this system, the cytotoxicity of DOX kills the tumor and the KGFRWR peptide inhibits the MMP activity (Ji et al., 2018). Likewise, nanoparticles metallofullerenol Gd@C82(OH)22 can block MMP-2 and MMP-9 synthesis through allosteric inhibition (Kang et al., 2012). Nanocarriers such as lysolipid-containing thermosensitive liposomes deliver marimastat (Lyu et al., 2019). Likewise, peptide nanofibers conjugated with siRNA or shRNA MMPs could effectively effective target MMPs (Mazza et al., 2019). Another nanoplatform consisting of HPAA-MTX/shMMP-9 (cationic hyperbranched poly (amido amine) (HPAA) with MTX and shMMP-9 plasmid) could significantly reduce the tumor growth in MCF 7 tumor-bearing animal as well as the decrease in the invasiveness and apoptosis induction in nasopharyngeal carcinoma HNE-1 cells (Tang et al., 2018; Liu et al., 2019).

## Conclusion

Remodeling and degradation of ECM are crucial events in metastasis and MMPs; a family of zinc-dependent proteases controls this process and promotes the progression of tumors into the distant site. MMPs play an important role in the inflammatory process and are known to influence the onset and progression of many cancer cases. Furthermore, MMPs play an important role in angiogenesis and cancer growth. MMPs play an important role in precision medicine because they can act as biomarkers. According to this review, investigating diverse classes of MMPs is critical in understanding their involvement in cancer progression and becoming therapeutic targets in cancer therapies. MMPs also are thought to promote the growth of the tumor cells once they have metastasized. In addition to ECM degradation, they are involved in the activation of cell surface proteins and the shedding of membrane-bound receptor molecules, regulating growth factors and chemokines. Moreover, inflammation, migration, and invasion of tumor cells and tumor-specific angiogenesis are also regulated by MMPs. MMPs are involved at various levels, during transcription, translation, and zymogen activation and by the activity of its endogenous inhibitor, TIMPs. TIMPs bind MMPs in a stoichiometric 1:1 ratio and thereby block access of substrates to the catalytic domain of the endopeptidases. An imbalance between active MMPs and TIMPs, favoring MMP activity can lead to ECM degradation, whereas favoring TIMPs leads to ECM deposition. Hence, MMPs is a novel target for cancer therapy, and several agents based on small molecule inhibitors, monoclonal antibody, and nanoparticles have been developed to improve patient survival.
